# Oxidative-Stress Biomarkers and Pathologic Response to Neoadjuvant Chemoradiotherapy in Locally Advanced Rectal Cancer: A Prospective Cohort Study

**DOI:** 10.3390/cancers18121939

**Published:** 2026-06-14

**Authors:** Hayriye Şahinli, Galip Can Uyar, Yakup Düzköprü, Özlem Aydın İsak, Ayşe Arzu Eren, Salim Neşelioğlu

**Affiliations:** 1 Department of Medical Oncology, Ankara Etlik City Hospital, 06170 Ankara, Turkey; g.can_uyar@hotmail.com (G.C.U.); ozlemaydin_87@hotmail.com (Ö.A.İ.); 2 Department of Medical Oncology, Aksaray State Hospital, 68100 Aksaray, Turkey; yakupduzkopru@gmail.com; 3 Department of Biochemistry, Ankara Etlik City Hospital, 06170 Ankara, Turkey; aerenst@gmail.com; 4 Department of Biochemistry, Ankara Yıldırım Beyazıt University, 06370 Ankara, Turkey; salim_neselioglu@hotmail.com

**Keywords:** chemoradiotherapy, ischemia-modified albümin (IMA), oxidative stress, rectal cancer, tumor regression

## Abstract

**Background**: Response to neoadjuvant chemoradiotherapy (CRT) in locally advanced rectal cancer (LARC) varies considerably, and oxidative stress may modulate radiosensitivity. This study evaluated ischemia-modified albumin (IMA) and thiol–disulfide homeostasis as potential biochemical predictors of pathological tumor regression. **Methods**: A prospective observational cohort study was conducted to assess pre- and post-treatment oxidative stress biomarkers in patients with LARC receiving capecitabine-based long-course CRT. Serum IMA, native thiol, total thiol, and disulfide levels were quantified spectrophotometrically. Pathologic regression was graded according to the Modified Ryan system as good (TRG 0–1) or poor (TRG 2–3). Receiver operating characteristic (ROC) analyses, Firth-penalized logistic regression, and internal validation using cross-validation, calibration, and decision-curve analyses were performed. **Results**: Of 38 screened patients, 31 met eligibility criteria and completed CRT, alongside 31 matched healthy controls. Compared with controls, patients had higher baseline disulfide (15.7 ± 5.2 vs. 11.9 ± 3.1 µmol/L; *p* = 0.012) and IMA levels (0.886 ± 0.062 vs. 0.798 ± 0.048 ABSU; *p* = 0.006). Poor responders exhibited higher pre-treatment IMA (0.927 ± 0.045 vs. 0.842 ± 0.050 ABSU; *p* = 0.020) and disulfide levels (18.4 ± 5.2 vs. 13.0 ± 3.8 µmol/L; *p* = 0.012). Pre-treatment IMA demonstrated the highest predictive accuracy for poor tumor regression (AUC = 0.872; 95% CI 0.751–0.993). In multivariable Firth-penalized logistic regression, elevated baseline IMA was independently associated with poor pathological response (OR = 3.63; 95% CI 1.22–16.20; *p* = 0.043), whereas negative circumferential resection margin (CRM) status was independently associated with favorable regression (OR = 0.21; 95% CI 0.02–0.71; *p* = 0.003). The internally validated model demonstrated excellent discrimination (AUC = 0.948; 95% CI 0.866–0.966) and good calibration. **Conclusions**: Baseline IMA and CRM status were independently associated with pathological response after CRT in LARC. These findings suggest that oxidative-stress biomarkers may have potential value for response stratification; however, the results should be considered exploratory and require external validation in larger independent cohorts before clinical application.

## Simple Summary

Patients with locally advanced rectal cancer can respond very differently to chemoradiotherapy given before surgery. Currently, clinicians have limited tools to predict which patients will achieve a favorable tumor response. Oxidative stress, a biological process caused by an imbalance between harmful molecules and the body’s defense mechanisms, may influence how tumors respond to treatment. In this prospective study, we evaluated blood-based oxidative stress markers, including ischemia-modified albumin and thiol–disulfide balance, in patients receiving preoperative chemoradiotherapy. We found that higher levels of ischemia-modified albumin before treatment were associated with poorer tumor regression after therapy. These findings suggest that simple blood tests reflecting oxidative stress may help identify patients who are less likely to respond well to treatment. Although the results require confirmation in larger studies, they provide preliminary evidence that oxidative stress biomarkers could contribute to more individualized treatment strategies for rectal cancer.

## 1. Introduction

Locally advanced rectal cancer (LARC) remains a major oncologic challenge, characterized by high rates of local recurrence and variable responses to multimodal therapy. Neoadjuvant chemoradiotherapy (CRT) followed by total mesorectal excision has become the standard of care, significantly improving local control and sphincter preservation rates [1,2,3]. However, tumor response to CRT exhibits marked heterogeneity, ranging from complete pathological remission to minimal regression. Accurate prediction of therapeutic response before treatment is crucial to optimize patient selection for organ preservation strategies, minimize overtreatment, and improve long-term outcomes [4,5].

Pathological tumor regression grade (TRG) after CRT is among the strongest prognostic indicators for local control and survival [5,6,7]. Numerous clinicopathologic and molecular predictors, such as baseline T/N stage, carcinoembryonic antigen (CEA) levels, and microsatellite instability (MSI), have been investigated, yet none have achieved reliable predictive accuracy across diverse populations [8,9]. Moreover, recent advances in total neoadjuvant therapy (TNT), a treatment strategy in which all planned systemic chemotherapy and radiotherapy are delivered before surgery, have further emphasized the biological complexity of treatment resistance, suggesting that traditional clinical and anatomical parameters fail to capture the multifactorial determinants of treatment response. Consequently, identifying novel, dynamic, and biologically grounded biomarkers remains an unmet need in rectal cancer management.

Emerging evidence highlights the pivotal role of oxidative stress and redox imbalance in modulating tumor response to radiotherapy. Ionizing radiation generates reactive oxygen species (ROS), which induce DNA damage and apoptosis, but persistent oxidative stress can promote radioresistance through hypoxia-inducible and antioxidant response pathways [10,11]. Among circulating oxidative markers, ischemia-modified albumin (IMA) reflects structural alteration of the albumin N-terminus under oxidative or ischemic conditions and serves as an integrative indicator of systemic redox status [12]. In parallel, dynamic thiol–disulfide homeostasis represents a key mechanism for maintaining redox balance and regulating cellular defense against oxidative injury. Previous studies in gastrointestinal malignancies have demonstrated altered IMA and thiol–disulfide levels compared with healthy controls [13,14]; however, their predictive value for pathological response in rectal cancer, particularly within the neoadjuvant setting, remains insufficiently defined.

Given these mechanistic and clinical considerations, oxidative-stress biomarkers have emerged as promising candidates for response prediction in LARC. Based on these considerations, we conducted a prospective, dual-center observational study to investigate the prognostic relevance of oxidative-stress biomarkers, including IMA, native thiol, total thiol, and disulfide levels, in patients with LARC undergoing neoadjuvant CRT. We hypothesized that elevated baseline IMA and altered thiol–disulfide dynamics would be associated with poor pathological tumor regression and that integrating these biochemical markers with clinicopathologic features could enhance prediction of therapeutic response in LARC.

## 2. Materials and Methods

### 2.1. Study Design and Population

This prospective observational study was conducted between January 2021 and December 2023 at the Medical Oncology Department of Ankara Dışkapı Yıldırım Beyazıt Training and Research Hospital, which was relocated to Ankara Etlik City Hospital in November 2022. A total of 31 patients with histologically confirmed LARC who were scheduled to receive neoadjuvant CRT were enrolled. Additionally, 31 age- and sex-matched healthy volunteers without chronic diseases or medication use affecting oxidative balance were recruited as the control group, each providing a single fasting venous blood sample.

Eligible patients were ≥18 years old, had LARC (stage II–III), and an Eastern Cooperative Oncology Group (ECOG) performance status of 0–2 [15]. Exclusion criteria included distant metastasis, severe hepatic, renal, or thyroid dysfunction, autoimmune or rheumatologic disorders, or concurrent use of anti-inflammatory or immunosuppressive drugs.

Demographic and clinical data, including age, sex, body mass index (BMI, calculated as weight in kilograms divided by height in meters squared), smoking history, diabetes mellitus, hypertension, medication use (e.g., metformin, antihypertensives), and comorbidity burden quantified by the modified Charlson Comorbidity Index (mCCI) [16], were recorded. Baseline clinicopathologic variables, including tumor location, clinical T and N stages, extramural venous invasion (EMVI), and circumferential resection margin (CRM) status were retrieved from pre-treatment magnetic resonance imaging (MRI) reports and pathology archives. Baseline laboratory parameters, including serum albumin, CEA, and carbohydrate antigen 19-9 (CA19-9), were collected from hospital records.

### 2.2. Treatment and Pathologic Evaluation

All patients received standard long-course neoadjuvant CRT, consisting of capecitabine-based chemotherapy administered concurrently with 50.4 Gy radiotherapy delivered in 28 fractions. Surgical resection was performed approximately 6–8 weeks after CRT completion. Pathologic tumor regression was assessed according to the Modified Ryan TRG system [17]. For analytical purposes, patients were categorized as good responders (TRG 0–1) or poor responders (TRG 2–3), consistent with previous studies demonstrating that complete or near-complete tumor regression is associated with more favorable oncologic outcomes after neoadjuvant treatment for rectal cancer [5,17].

### 2.3. Follow-Up and Survival Evaluation

Patients were followed every 3 months for the first 2 years and every 6 months thereafter with physical examination, laboratory testing, and imaging (pelvic MRI or CT, thoracoabdominal CT). Disease-free survival (DFS) was defined as the time from surgery to the first documented recurrence or death from any cause.

Overall survival (OS) was defined as the time from diagnosis to death from any cause or last follow-up. Survival outcomes were estimated using the Kaplan–Meier method; however, due to the limited number of events and absence of median survival, no log-rank or Cox proportional hazards analyses were performed.

### 2.4. Sample Collection and Biochemical Analysis

Peripheral venous blood samples were collected between 08:00 and 10:00 a.m. after an overnight fast of at least 8 h to minimize diurnal and nutritional variability. For patients, samples were obtained one week before initiation of neoadjuvant CRT (pre-treatment) and within one week after completion of CRT (post-treatment).

Healthy controls provided a single fasting morning blood sample during routine laboratory evaluation, collected contemporaneously with the patient pre-treatment period. All blood samples were obtained during routine clinical visits without additional invasive procedures. Samples were centrifuged at 1500× *g* for 10 min, and serum aliquots were stored at −80 °C until analysis.

Serum native thiol (NT), total thiol (TT), and disulfide (DS) levels were quantified using the fully automated spectrophotometric method developed by Erel and Neşelioğlu [18], which dynamically evaluates thiol–disulfide homeostasis. In this assay, dynamic disulfide bonds are reduced to free thiol groups with sodium borohydride (NaBH_4_); residual NaBH_4_ is neutralized with formaldehyde, and thiols react with 5,5′-dithiobis-(2-nitrobenzoic acid) to produce measurable chromophores.

The disulfide concentration was calculated as (TT − NT) /2, and derived ratios (DS/TT, DS/NT, and NT/TT) were computed to assess redox balance. Serum IMA levels were determined using the Bar-Or colorimetric method [12] and expressed in absorbance units (ABSU) on a Roche-Hitachi Cobas c501 (Ibaraki, Japan) automated analyzer. Serum albumin concentrations (g/dL) were simultaneously measured by the bromocresol green (BCG) method.

All analyses were performed by blinded laboratory personnel in the central biochemistry laboratory. Institutional authorization for sample processing and storage was granted by the Ankara Etlik City Hospital Biochemistry and Genomics Laboratory (BGOF).

### 2.5. Ethical Considerations

The study was approved by the Ethics Committee of Ankara Dışkapı Yıldırım Beyazıt Training and Research Hospital (decision no. 61/27, dated 25 March 2019) and conducted in accordance with the principles of the Declaration of Helsinki and Good Clinical Practice (GCP) guidelines. Written informed consent was obtained from all participants before enrollment.

### 2.6. Statistical Analysis

All statistical analyses were performed using SPSS version 25.0 (IBM Corp., Armonk, NY, USA) and R version 4.5.2 (The R Foundation for Statistical Computing, Vienna, Austria). Continuous variables were expressed as mean ± standard deviation (SD) or median (interquartile range, IQR), depending on data distribution, while categorical variables were summarized as frequencies and percentages (%). Normality was assessed with the Shapiro–Wilk test. Between-group comparisons were performed using the Mann–Whitney U test or independent-samples *t*-test for continuous variables, and the χ^2^ test or Fisher’s exact test for categorical variables, as appropriate. Within-patient changes in oxidative stress markers (pre- vs. post-treatment) were evaluated using paired *t*-tests or Wilcoxon signed-rank tests.

Receiver operating characteristic (ROC) curve analyses were conducted to evaluate the predictive performance of each oxidative stress biomarker for pathologic tumor regression (TRG 0–1 vs. 2–3). The area under the curve (AUC) and 95% confidence intervals (CIs) were calculated using DeLong’s method. Optimal cut-off values were derived using Youden’s J statistic (J = sensitivity + specificity − 1), and diagnostic metrics including sensitivity, specificity, positive predictive value (PPV), negative predictive value (NPV), and overall accuracy were reported.

Variables with *p* < 0.10 in univariable analysis were entered into multivariable Firth-penalized logistic regression, implemented using the logistf package version 1.26.1 in R version 4.5.2 (R Foundation for Statistical Computing, Vienna, Austria), to account for small-sample bias and data separation. Model estimates were presented as odds ratios (ORs) with 95% CIs. Multicollinearity was assessed using variance inflation factors (VIFs), and predictors with VIF > 5 were excluded. Model refinement was guided by parsimony and clinical interpretability.

Internal validation of the final model was performed using both leave-one-out cross-validation (LOOCV) and bootstrap resampling (1000 iterations) to obtain optimism-corrected AUC and calibration parameters. Calibration performance was assessed by intercept, slope, Brier score, and calibration plot generated using the rms package version 8.1-1 in R version 4.5.2. Discrimination was evaluated using the AUC, whereas clinical utility was assessed using decision curve analysis (DCA), which compared the net benefit of the model with those of the “treat-all” and “treat-none” strategies across threshold probabilities ranging from 0.1 to 0.8.

For survival endpoints, DFS and OS were estimated using the Kaplan–Meier method. Owing to the limited number of events and the absence of median survival, no group comparisons or Cox proportional hazards modeling were performed.

All statistical tests were two-sided, and *p* < 0.05 was considered statistically significant.

## 3. Results

**Figure 1:**
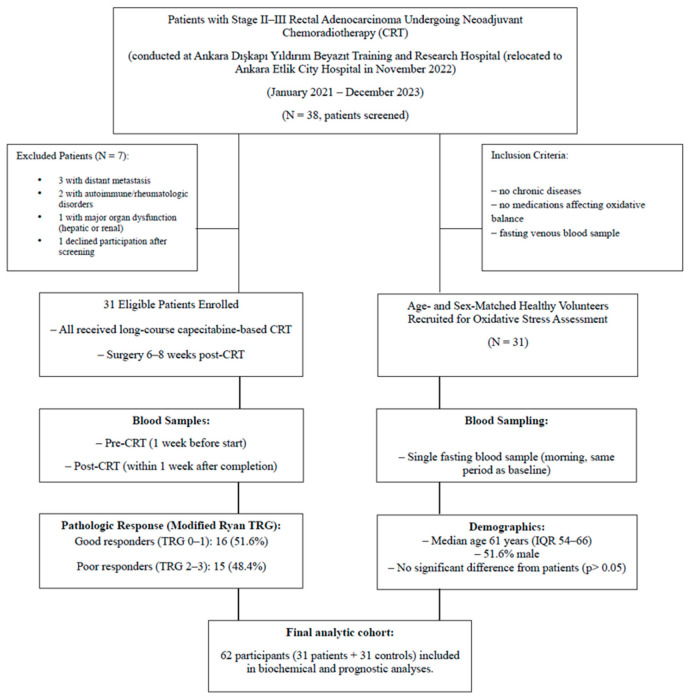
Study flow diagram of the prospective dual-center cohort. Abbreviations: CRT = chemoradiotherapy; IQR = interquartile range; TRG = tumor regression grade. Caption: Flow diagram showing patient enrollment, exclusion criteria, and final analytic cohort formation. Among 38 screened patients with stage II–III rectal adenocarcinoma evaluated for neoadjuvant chemoradiotherapy (CRT), 7 were excluded (due to distant metastasis, autoimmune/rheumatologic disorders, or organ dysfunction), leaving 31 eligible participants who all received long-course capecitabine-based CRT. Peripheral blood samples were collected at two time points: pre-CRT (one week before treatment) and post-CRT (within one week after completion). Thirty-one age- and sex-matched healthy volunteers (median age 61 years [IQR 54–66]; 51.6% male) provided a single fasting blood sample during the same period for oxidative stress assessment. The final analytic cohort consisted of 62 participants (31 patients and 31 controls) included in biochemical and prognostic analyses.

Between January 2021 and December 2023, 38 patients with stage II–III rectal adenocarcinoma were screened for eligibility. Seven patients were excluded due to distant metastasis, autoimmune or rheumatologic disease, or major organ dysfunction, leaving 31 participants who completed long-course capecitabine-based CRT followed by total mesorectal excision (Figure 1).

Thirty-one patients with rectal adenocarcinoma (median age = 62 years [IQR 55–69]; 58.1% male) and 31 age- and sex-matched healthy controls (median age = 61 years [IQR 54–66]; 51.6% male) were analyzed, with no significant differences in age (*p* = 0.785) or sex distribution (*p* = 0.849).

At a median follow-up of 28.6 months (IQR 22.4–35.8), six patients (19.4%) developed recurrence and one (3.2%) died. Median DFS and OS were not reached; the estimated 2-year DFS and OS rates were 78.5% (95% CI 62.1–95.0) and 93.5% (95% CI 84.0–100), respectively. Given the limited number of events, these survival outcomes should be interpreted descriptively.

ECOG performance status was ≤ 1 in 26 (83.9%) patients and 2 in 5 (16.1%). Diabetes mellitus and hypertension were present in 7 (22.6%) and 8 (25.8%) patients, respectively. A mCCI ≥ 4 was observed in 21 (67.7%) patients. Clinical T stage was T3–4 in 19 (61.3%) patients, while 28 (90.3%) had clinically positive nodes. EMVI and CRM positivity were detected in 16 (51.6%) and 14 (45.2%) patients, respectively. Baseline hypoalbuminemia (<35 g/L) was observed in 7 (22.6%) patients. A TRG 0–1 was achieved in 16 (51.6%) patients, whereas 15 (48.4%) showed a TRG 2–3. Baseline clinical and pathological characteristics are summarized in Table 1.

Compared with healthy controls, patients with rectal cancer exhibited significantly higher baseline disulfide levels (15.7 ± 5.2 µmol/L vs. 11.9 ± 3.1 µmol/L, *p* = 0.012) and IMA concentrations (0.886 ± 0.062 ABSU vs. 0.798 ± 0.048 ABSU, *p* = 0.006), whereas native and total thiol fractions showed no significant differences between groups (all *p* > 0.05). Among patients, pre-treatment disulfide and IMA levels were significantly higher in poor pathological responders than in good responders (18.4 ± 5.2 µmol/L vs. 13.0 ± 3.8 µmol/L, *p* = 0.012; 0.927 ± 0.045 ABSU vs. 0.842 ± 0.050 ABSU, *p* = 0.020). Post-treatment IMA levels remained elevated in the poor-responder group (0.860 ± 0.041 ABSU vs 0.808 ± 0.052 ABSU, *p* = 0.031). No other thiol fractions or redox indices demonstrated significant associations with tumor regression (*p* > 0.05). Detailed biochemical comparisons are shown in Table 2.

Oxidative stress biomarkers according to pathological tumor regression are summarized in Table 3. Pre-treatment disulfide levels were significantly higher in poor responders compared with good responders (18.4 ± 5.2 µmol/L vs. 13.0 ± 3.8 µmol/L, *p* = 0.012). Similarly, pre-treatment IMA values were elevated in the poor-response group (0.927 ± 0.045 ABSU vs. 0.842 ± 0.050 ABSU, *p* = 0.020). After treatment, IMA levels remained higher among poor responders (0.860 ± 0.041 ABSU vs. 0.808 ± 0.052 ABSU, *p* = 0.031). No significant differences were observed between groups for native thiol, total thiol, or derived redox ratios such as disulfide/native thiol, disulfide/total thiol, or native/total thiol (all *p* > 0.05).

### 3.1. Diagnostic Performance of Oxidative Stress Biomarkers

**Figure 2:**
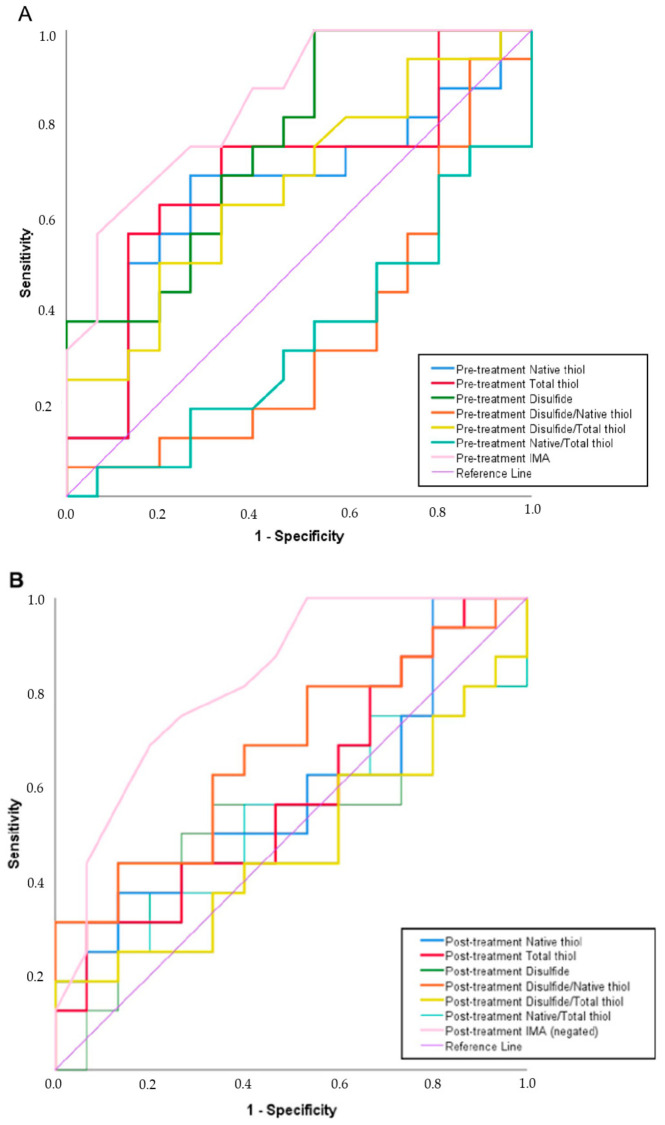

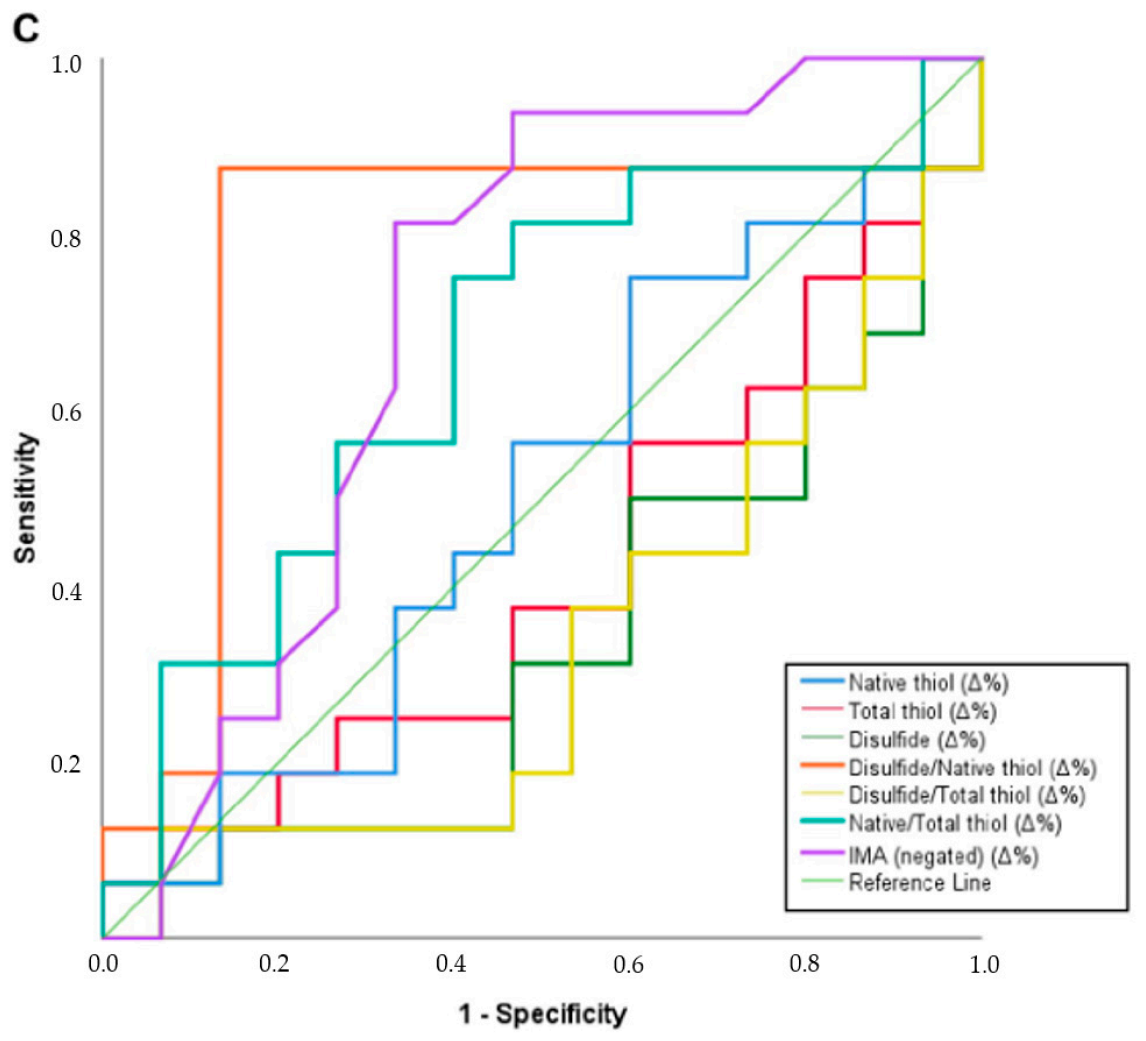
(**A**–**C**). Receiver operating characteristic (ROC) curves illustrating the discriminative ability of thiol/disulfide homeostasis and ischemia-modified albumin (IMA) parameters for predicting pathological treatment response in patients with rectal cancer. (**A**) Pre-treatment, (**B**) post-treatment, and (**C**) percentage-change (Δ%) levels of serum native thiol, total thiol, disulfide, disulfide/native thiol ratio, disulfide/total thiol ratio, native/total thiol ratio, and IMA are shown. AUC values with 95% confidence intervals are summarized in Table 4.

ROC curve analyses were performed to evaluate the discriminative ability of oxidative stress parameters for predicting pathological tumor regression. Among all evaluated biomarkers, pre-treatment IMA demonstrated the highest area under the curve (AUC = 0.872, 95% CI 0.751–0.993), followed by disulfide level (AUC = 0.807, 95% CI 0.661–0.954). At the optimal cut-off values determined by Youden’s J index, IMA ≥ 0.89 ABSU achieved 81.3% sensitivity and 80.0% specificity, whereas disulfide ≥ 15.6 µmol/L yielded 73.3% sensitivity and 75.0% specificity. The diagnostic performance metrics of all biomarkers are summarized in Table 4, and the corresponding ROC curves are illustrated in Figure 2A–C.

Notably, pre-treatment IMA (AUC = 0.846, *p* = 0.001), post-treatment IMA (AUC = 0.825, *p* = 0.002), and ΔDisulfide/Native thiol ratio (AUC = 0.788, *p* = 0.006) demonstrated the highest discriminative performance for good vs. poor pathological response.

### 3.2. Predictors of Pathologic Tumor Regression

In univariable analyses, several clinical and biochemical parameters were significantly associated with pathological tumor regression. Lower baseline albumin (OR = 6.43, 95% CI 1.03–21.12, *p* = 0.025), higher pre-treatment disulfide (OR = 7.13, 95% CI 1.36–21.30, *p* = 0.010), and elevated pre-treatment IMA levels (OR = 6.00, 95% CI 1.26–28.55, *p* = 0.020) were associated with TRG 2–3. Among tumor-related factors, advanced clinical T stage (OR = 0.03, 95% CI 0.003–0.32, *p* = 0.001), EMVI (OR = 0.12, 95% CI 0.06–0.53, *p* < 0.001), and CRM positivity (OR = 0.16, 95% CI 0.05–0.39, *p* < 0.001) also demonstrated significant associations.

In multivariable Firth-penalized logistic regression, higher baseline IMA levels remained independently associated with poor pathological response (OR = 3.63, 95% CI 1.22–16.20, *p* = 0.043), whereas negative CRM status was independently associated with good pathological response (OR = 0.21, 95% CI 0.02–0.71, *p* = 0.003). Detailed univariable and multivariable regression results are summarized in Table 5.

### 3.3. Model Performance and Validation

**Figure 3:**
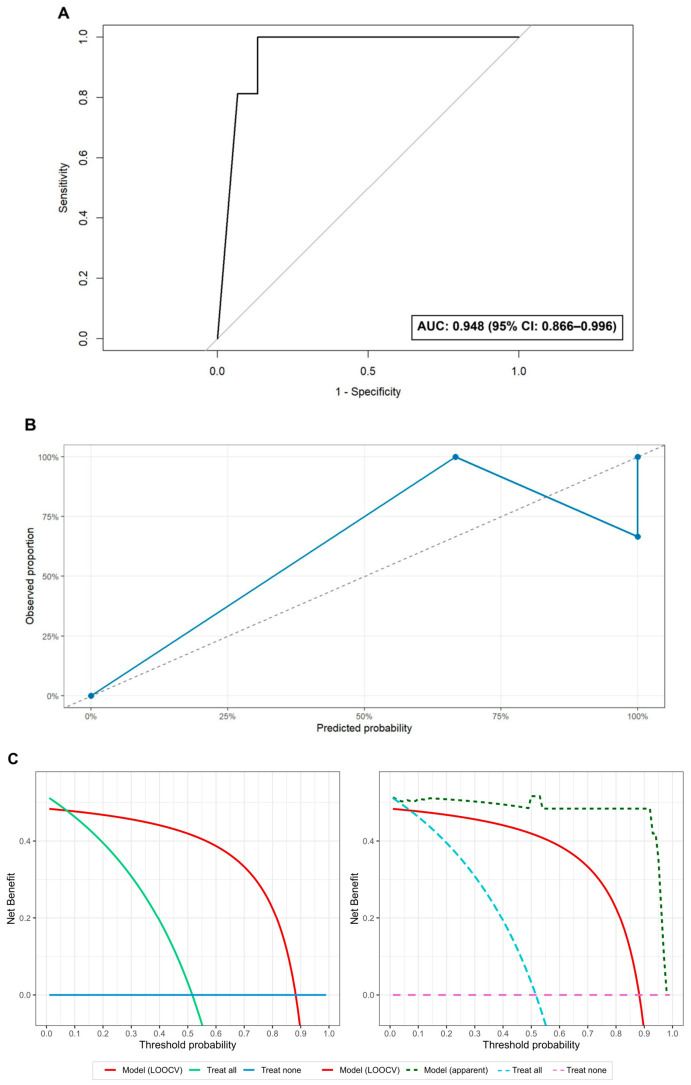
(**A**–**C**). Discrimination, calibration, and clinical utility of the simplified Firth logistic regression model. Abbreviations: AUC = area under the curve; DCA = decision curve analysis; LOOCV = leave-one-out cross-validation; ROC = receiver operating characteristic. (**A**) Receiver operating characteristic (ROC) curve demonstrating excellent discrimination, with an area under the curve (AUC) of 0.948 (95% CI: 0.866–0.996) based on leave-one-out cross-validation (LOOCV). (**B**) Calibration plot showing good agreement between predicted and observed probabilities (slope = 0.07; intercept = −0.26; Brier score = 0.097), indicating minimal underestimation in higher predicted ranges. (**C**) Decision curve analyses (DCA) of the simplified Firth logistic regression model. The left panel illustrates the leave-one-out cross-validation (LOOCV)-corrected model, which consistently outperformed both “treat-all” and “treat-none” strategies across a wide range of threshold probabilities (0.1–0.8). The right panel compares the apparent and LOOCV-corrected models, showing only minimal optimism correction and confirming the model’s stable net benefit profile, indicating robust internal validity and favorable clinical applicability.

The LOOCV-validated Firth logistic regression model demonstrated excellent discrimination for predicting pathological treatment response, achieving an area under the ROC curve (AUC = 0.948, 95% CI 0.866–0.966) (Figure 3A).

Calibration analysis indicated close agreement between predicted and observed probabilities (Figure 3B). DCA showed that the model provided greater net benefit than both “treat-all” and “treat-none” strategies across threshold probabilities ranging from 0.1 to 0.8 (Figure 3C). However, given the limited sample size and absence of external validation, these findings should be considered exploratory.

## 4. Discussion

In this prospective, dual-center observational study, we evaluated oxidative stress dynamics in patients with LARC undergoing neoadjuvant CRT and identified a marked systemic redox imbalance compared with healthy controls. Serum IMA and disulfide concentrations were significantly elevated in patients, indicating increased oxidative burden even before treatment initiation. Given potential differences in inflammation, nutritional status, comorbidity burden, and medication exposure, the healthy-control comparison should be interpreted cautiously and primarily as contextual evidence of altered systemic redox homeostasis. In univariable analyses, higher baseline IMA and disulfide levels were associated with poorer pathological tumor regression; however, in multivariable Firth logistic regression, baseline IMA emerged as an independent factor associated with unfavorable pathological response. Negative CRM status was independently associated with favorable response. Although the internally validated model demonstrated encouraging discriminatory performance, the findings should be interpreted cautiously given the limited sample size and the absence of external validation. Collectively, these results suggest that elevated baseline IMA, reflecting persistent oxidative stress and disrupted redox homeostasis, may be associated with poorer response to CRT in LARC.

Growing evidence suggests that oxidative stress plays a critical role in modulating tumor radiosensitivity and therapeutic response in rectal and colorectal cancers. IMA, generated through N-terminal modification of albumin under oxidative or ischemic conditions, is recognized as a comprehensive indicator of systemic redox imbalance [12]. Previous studies have demonstrated increased oxidative stress and altered redox homeostasis in colorectal malignancies. Ellidag et al. reported significantly elevated IMA levels in patients with colorectal cancer compared with healthy controls [19]. Similarly, Bilgin et al. demonstrated disturbances in dynamic thiol–disulfide homeostasis in colorectal cancer, supporting the presence of systemic redox dysregulation [20]. Furthermore, Özdemir et al. observed elevated IMA levels in colorectal cancer, whereas thiol–disulfide parameters showed no significant differences compared with controls, highlighting the biological heterogeneity of oxidative-stress biomarkers [21]. In addition, Serbanescu et al. reported oxidative-stress alterations during neoadjuvant CRT in patients with LARC [22]. Consistent with these observations, our prospective study, with dynamic pre- and post-CRT assessments and a parallel healthy-control cohort, showed markedly higher IMA levels in patients and identified baseline IMA as an independent predictor of poor pathological tumor regression. Disulfide concentrations, reflecting increased oxidative load, were also elevated and associated with poor response in univariable analyses, consistent with prior reports [20,22,23]. However, their independent prognostic value did not persist in multivariable analysis, likely owing to biological and clinical variability among cohorts. Thiol fractions and their derived ratios (NT/TT, DS/NT, DS/TT) likewise demonstrated inconsistent clinical associations, potentially reflecting their short half-life and susceptibility to inflammation, nutritional status, hepatic function, and circulating albumin levels. Although the automated spectrophotometric assay developed by Erel and Neşelioğlu [18] minimizes analytical variability, thiol-related markers may be less stable than IMA in capturing cumulative oxidative stress. Taken together, our prospective design incorporating longitudinal measurements and a healthy-control comparison strengthens the reliability of these findings. Within the heterogeneous body of literature, IMA may represent a relatively stable biomarker of systemic oxidative stress associated with pathological response to CRT. Elevated baseline IMA may reflect a pro-oxidative milieu characterized by impaired redox homeostasis, hypoxia-related signaling, and adaptive antioxidant responses linked to treatment resistance [24]. However, IMA is not tumor-specific and may also be influenced by systemic inflammation, nutritional status, hepatic function, circulating albumin levels, and other host-related factors. Therefore, IMA should be interpreted as a candidate biomarker reflecting oxidative-stress burden rather than a direct measure of intrinsic tumor radiosensitivity. In contrast, thiol–disulfide parameters demonstrated greater biological variability and require further validation in larger prospective cohorts.

In our study, among the clinicopathologic variables evaluated, only negative CRM status remained independently associated with favorable pathological tumor regression. This finding aligns with previous evidence indicating that CRM negativity following neoadjuvant CRT is one of the strongest prognostic factors for both local control and long-term survival in LARC. Nagtegaal et al. [25] demonstrated that CRM positivity markedly increases the risk of local recurrence, while the ESMO guidelines [3] emphasize the close association between CRM negativity, favorable tumor regression, and lower local recurrence risk. Similarly, in the “watch-and-wait” series reported by Habr-Gama et al. [26], most patients achieving complete clinical response exhibited CRM negativity. Collectively, these findings suggest that CRM status primarily reflects local tumor extent, mesorectal fascia involvement, and anatomical resectability rather than intrinsic tumor radiosensitivity. Therefore, the association between CRM negativity and favorable pathological regression may be explained by less advanced local disease and more favorable anatomical characteristics, both of which may facilitate improved response to CRT. In contrast, other classical parameters such as age, sex, tumor location, and baseline T/N stage showed no significant association with pathological response. This observation is consistent with multiple prospective cohorts in the literature. Armstrong et al. [27] and Zhang et al. [28] likewise reported that conventional clinicopathologic factors, including age, sex, tumor location, and baseline CEA levels, carry limited predictive value for achieving pathological complete response after CRT. These findings reinforce the concept that therapeutic outcomes in rectal cancer are influenced by a complex interplay of clinicopathologic, anatomical, and biological factors, including redox homeostasis, hypoxia-related signaling, immune modulation, and DNA-damage repair. Our results suggest that oxidative-stress-related biomarkers may provide complementary biological information beyond conventional clinicopathologic parameters and warrant further investigation in larger prospective studies. Nevertheless, the lack of statistical significance for several clinicopathologic variables could be partly attributed to patient heterogeneity, the relatively small sample size, and the observational design of our cohort. Future large-scale, multicenter studies are warranted to validate these findings and further clarify the prognostic value of both conventional clinical factors and oxidative-stress-related biomarkers.

The present investigation highlights the potential of oxidative-stress biomarkers as biochemical predictors of treatment response in LARC, bridging metabolic redox imbalance with pathological regression. While these findings expand the biochemical understanding of neoadjuvant response, redox homeostasis is also modulated by systemic host factors such as body composition, inflammation, and nutritional reserve [23,29]. Integrating these dimensions could improve the biological interpretability and clinical utility of redox-based predictive models. In addition to IMA and thiol, disulfide parameters, other oxidative-stress markers, such as malondialdehyde (MDA), total antioxidant capacity (TAC), superoxide dismutase (SOD), catalase (CAT), and glutathione peroxidase (GPx), have been investigated in colorectal and rectal cancer cohorts. Elevated MDA levels and reduced SOD and GPx activities have been associated with enhanced lipid peroxidation and impaired antioxidant defense, correlating with advanced disease and poorer therapeutic response [13,14]. More recently, transcriptomic analyses have identified oxidative stress-related gene signatures linked to tumor immunity and prognosis in colorectal cancer [30], further underscoring the multifaceted role of redox imbalance in tumor biology. However, these enzyme-based or gene-expression markers often exhibit limited reproducibility due to short half-life, interindividual variability, and laboratory dependence. By contrast, IMA represents a more stable, albumin-derived indicator of cumulative oxidative stress, maintaining clinical interpretability even in single-sample assessments. This methodological robustness may partly explain why IMA retained independent predictive significance in the present cohort, whereas other oxidative markers reported in previous studies have yielded inconsistent prognostic value. Recent evidence, including the CINR-AI study [31], has shown that post-treatment sarcopenia, elevated C-reactive protein/albumin ratio (CAR), and high systemic immune-inflammation index (SII) independently predict poor tumor regression in patients receiving total neoadjuvant therapy (TNT). These observations support the mechanistic link between oxidative stress, muscle catabolism, and chronic inflammation. Incorporating quantitative muscle indices together with nutritional–inflammatory scores such as the CAR, neutrophil-to-lymphocyte ratio (NLR), platelet-to-lymphocyte ratio (PLR), and SII may therefore capture the systemic repercussions of redox imbalance more comprehensively [31,32,33,34,35]. Additional exploration of lipid metabolism and hypoxia-regulated redox pathways (HIF-1α, Nrf2/KEAP1) could further refine mechanistic insight [36,37,38,39]. Future studies integrating biochemical, radiomic, and clinicopathologic variables into multimodal predictive models may further improve individualized response prediction in LARC [31,40,41,42].

This prospective study evaluated oxidative-stress biomarkers as potential biochemical indicators of pathological tumor regression in rectal cancer through systematic pre- and post-treatment blood sampling and the inclusion of a healthy control group. All patients received concurrent capecitabine-based CRT, ensuring treatment homogeneity and enabling reliable interpretation of therapy-induced redox changes. All biochemical analyses were performed using a fully automated, spectrophotometric assay standardized according to the Erel and Neşelioğlu method [18], which ensures high analytical reproducibility and minimizes operator-dependent bias. These methodological features strengthen the validity of the findings and support the exploratory evaluation of oxidative-stress biomarkers as candidate indicators of treatment response. Several limitations should be acknowledged. The relatively small sample size may reduce statistical power and external generalizability. In addition, the study was conducted at a single center; therefore, validation in multicenter, randomized, and adequately powered trials is required. The absence of a TNT cohort limits the applicability of the results to contemporary treatment protocols. Moreover, key molecular parameters, including MSI status and RAS/BRAF mutations, as well as clinically relevant inflammatory and nutritional factors such as CRP, NLR, SII, body composition, and sarcopenia, were not prospectively assessed. These factors may influence treatment response and could potentially confound or modify the observed associations between oxidative-stress biomarkers and pathological regression, preventing a comprehensive evaluation of systemic and tumor-related determinants of redox imbalance. Furthermore, tissue-based validation analyses, including immunohistochemical assessment of oxidative-stress-related pathways, were not available. Future translational studies integrating circulating biomarkers with tissue-level molecular and immunohistochemical characterization may help clarify the biological mechanisms underlying the observed associations. As an observational analysis, causal inference cannot be established, and residual confounding cannot be excluded. Finally, inter-laboratory validation of the biochemical assays was not performed, which may affect external comparability of quantitative values.

## 5. Conclusions

In conclusion, dynamic oxidative-stress alterations may represent promising candidate biomarkers associated with treatment response in LARC. Given the limited sample size, the low number of survival events, and the exclusive inclusion of patients treated with conventional long-course CRT, these findings should be considered exploratory and hypothesis-generating. Future large-scale, multicenter studies, ideally including TNT-treated cohorts and integrating biochemical, molecular, nutritional, radiomic, and tissue-based mechanistic parameters, are required to externally validate these findings, clarify their prognostic significance, and determine their applicability within contemporary treatment pathways.

## Figures and Tables

**Table 1:** Clinical and characteristics of the study population according to pathological tumor regression.

Variable	Category	Poor Response (Ryan 2–3) (*n* = 15), (48.4%)	Good Response (Ryan 0–1) (*n* = 16), (51.6%)	Overall (*n* = 31), (%)
Age	<65 years	7 (46.7)	9 (56.3)	16 (51.6)
	≥65 years	8 (53.3)	7 (43.8)	15 (48.4)
Gender	Female	4 (26.7)	9 (56.3)	13 (41.9)
	Male	11 (73.3)	7 (43.8)	18 (58.1)
ECOG PS	0–1	11 (73.3)	15 (93.8)	26 (83.9)
	2	4 (26.7)	1 (6.3)	5 (16.1)
Diabetes mellitus	Absent	12 (80.0)	12 (75.0)	24 (77.4)
	Present	3 (20.0)	4 (25.0)	7 (22.6)
Metformin use	Absent	14 (93.3)	12 (75.0)	26 (83.9)
	Present	1 (6.7)	4 (25.0)	5 (16.1)
Hypertension drug use	Absent	12 (80.0)	11 (68.8)	23 (74.2)
	Present	3 (20.0)	5 (31.3)	8 (25.8)
Smoking history	Absent	9 (60.0)	7 (43.8)	16 (51.6)
	Present	6 (40.0)	9 (56.3)	15 (48.4)
Baseline BMI (kg/m^2^)	<22.5	4 (26.7)	4 (25.0)	8 (25.8)
	≥22.5	11 (73.3)	12 (75.0)	23 (74.2)
mCCI	<4	3 (20.0)	7 (43.8)	10 (32.3)
	≥4	12 (80.0)	9 (56.3)	21 (67.7)
Baseline albumin (g/L)	<35	6 (40.0)	1 (6.3)	7 (22.6)
	≥35	9 (60.0)	15 (93.8)	24 (77.4)
Clinical T stage	1–2	1 (6.7)	11 (68.8)	12 (38.7)
	3–4	14 (93.3)	5 (31.3)	19 (61.3)
Clinical N stage	Negative	2 (13.3)	1 (6.3)	3 (9.7)
	Positive	13 (86.7)	15 (93.8)	28 (90.3)
EMVI	Negative	1 (6.7)	14 (87.5)	15 (48.4)
	Positive	14 (93.3)	2 (12.5)	16 (51.6)
CRM	Negative	1 (6.7)	16 (100.0)	17 (54.8)
	Positive	14 (93.3)	0 (0.0)	14 (45.2)
Baseline CEA (ng/mL)	<5	7 (46.7)	8 (50.0)	15 (48.4)
	≥5	8 (53.3)	8 (50.0)	16 (51.6)
Baseline CA19-9 (U/mL)	<37	11 (73.3)	10 (62.5)	21 (67.7)
	≥37	4 (26.7)	6 (37.5)	10 (32.3)
RT–surgery interval	≥8 weeks	10 (66.7)	14 (87.5)	24 (77.4)
	<8 weeks	5 (33.3)	2 (12.5)	7 (22.6)

Abbreviations: BMI, body mass index; CA19-9, carbohydrate antigen 19-9; CEA, carcinoembryonic antigen; CRM, circumferential resection margin; ECOG PS, Eastern Cooperative Oncology Group performance status; EMVI, extramural venous invasion; mCCI, modified Charlson Comorbidity Index; RT, radiotherapy.

**Table 2:** Biochemical parameters according to pathological response (pre-treatment, post-treatment, and Δ% change values; ROC-based cut-offs).

Parameters	Pre (Patients) *(n* = 31)	Post (Patients) (*n* = 31)	Δ% (Median [IQR])	Healthy Controls (*n* = 31)	*p* (Pre vs. Healthy)	*p* (Post vs. Healthy)	*p* (Pre vs. Post)
Native thiol (µmol/L)	299.4 ± 52.8	286.0 ± 73.1	−3.00 [−22.40; +9.96]	317.6 ± 45.2	0.118	0.241	0.204
Total thiol (µmol/L)	330.7 ± 56.1	327.7 ± 61.1	+0.49 [−23.18; +12.64]	342.5 ± 47.8	0.241	0.412	0.672
Disulfide (µmol/L)	15.7 ± 5.2	17.2 ± 7.0	+23.69 [−14.46; +105.60]	11.9 ± 3.1	0.012	0.004	0.210
Disulfide/Native thiol (%)	6.14 ± 1.98	6.51 ± 2.13	+1.30 [−23.24; +46.25]	5.12 ± 1.02	0.058	0.072	0.286
Disulfide/Total thiol (%)	4.77 ± 1.48	5.25 ± 1.82	+24.03 [−31.34; +92.26]	3.91 ± 0.83	0.064	0.069	0.241
Native/Total thiol (%)	90.46 ± 2.96	89.50 ± 3.64	−2.30 [−6.32; +1.72]	91.85 ± 1.72	0.033	0.021	0.058
IMA (ABS U)	0.886 ± 0.062	0.835 ± 0.054	+11.90 [−1.49; +14.72] *	0.798 ± 0.048	0.006	0.041	0.031

Abbreviations: IMA, ischemia-modified albumin; Δ%, relative percentage change between pre- and post-treatment values Notes: *p*-values represent comparisons between poor (Ryan 2–3) and good (Ryan 0–1) pathological responders using χ^2^ tests for categorical ROC-based thresholds. For continuous parameters, group comparisons were performed using Mann–Whitney U or independent-samples *t*-tests as appropriate. * ΔIMA values were calculated using raw (non-negated) IMA values to ensure consistent directionality across measurements. For ROC and regression analyses, negated IMA values were used to maintain interpretative alignment with oxidative stress indices.

**Table 3:** Oxidative stress biomarkers according to pathological tumor regression grade.

Parameter (*n* = 31)	Good Response *(n* = 16) (Median [IQR])	Poor Response (*n* = 15) (Median [IQR])	*p*-Value
Pre-treatment			
Native thiol (µmol/L)	312.4 ± 48.2	286.1 ± 54.8	0.094
Total thiol (µmol/L)	341.2 ± 49.6	321.4 ± 58.7	0.118
Disulfide (µmol/L)	13.0 ± 3.8	18.4 ± 5.2	0.012
Disulfide/Native thiol (%)	5.76 ± 1.72	6.54 ± 1.96	0.210
Disulfide/Total thiol (%)	4.32 ± 1.12	4.93 ± 1.54	0.236
Native/Total thiol (%)	91.1 ± 2.8	89.8 ± 3.2	0.158
IMA (ABS U)	0.842 ± 0.050	0.927 ± 0.045	0.020
Post-treatment			
Native thiol (µmol/L)	294.2 ± 71.0	279.0 ± 68.5	0.284
Total thiol (µmol/L)	333.4 ± 58.2	322.2 ± 60.9	0.336
Disulfide (µmol/L)	16.1 ± 6.1	18.3 ± 7.8	0.265
Disulfide/Native thiol (%)	6.25 ± 2.01	6.66 ± 2.18	0.278
Disulfide/Total thiol (%)	4.93 ± 1.66	5.37 ± 1.98	0.302
Native/Total thiol (%)	89.9 ± 3.3	89.1 ± 3.7	0.420
IMA (ABS U)	0.808 ± 0.052	0.860 ± 0.041	0.031
Δ % change			
Δ Native thiol (%)	−3.2 [−12.1; +7.8]	−4.1 [−16.3; +9.9]	0.594
Δ Total thiol (%)	+0.6 [−10.4; +13.1]	−1.3 [−11.9; +11.7]	0.641
Δ Disulfide (%)	+23.5 [−10.6; +87.1]	+25.1 [−9.4; +92.4]	0.728
Δ (Native/Total thiol) (%)	−2.0 [−6.2; +1.7]	−2.5 [−6.8; +1.4]	0.582
Δ IMA (%)	+10.9 [+5.2; +14.7]	+12.4 [+9.1; +14.8]	0.649

Abbreviations: Δ%, relative percentage change between pre- and post-treatment values; IMA, ischemia-modified albumin; IQR, interquartile range; SD, standard deviation. Notes: Values are expressed as mean ± SD or median [IQR], as appropriate. Comparisons between good (Ryan 0–1) and poor (Ryan 2–3) pathological responders were performed using independent-samples *t*-tests or Mann–Whitney U tests. ΔIMA values were calculated using raw (non-negated) IMA measurements to preserve directional consistency across pre- and post-treatment assessments. For regression and ROC analyses, negated IMA values were used to align interpretability with other oxidative stress indices.

**Table 4:** Diagnostic performance of oxidative stress biomarkers for predicting pathological response.

Biomarker	AUC (95% CI)	*p*-Value	Cut-Off	Sensitivity (%)	Specificity (%)	PPV (%)	NPV (%)	Accuracy (%)
Pre-treatment								
Native thiol	0.654 (0.453–0.856)	0.144	245.05	81.3	80.0	79.0	82.0	80.6
Total thiol	0.687 (0.491–0.884)	0.075	264.15	87.5	80.0	82.3	85.2	83.9
Disulfide	0.758 (0.589–0.928)	0.014	11.40	93.8	73.3	77.8	92.0	83.9
Disulfide/Native thiol	0.346 (0.145–0.546)	0.144	5.21	62.5	56.7	58.0	61.0	59.7
Disulfide/Total thiol	0.665 (0.472–0.857)	0.118	4.05	68.8	60.0	63.2	66.0	64.5
Native/Total thiol	0.335 (0.143–0.528)	0.118	88.62	62.5	60.0	61.5	61.0	61.3
IMA	0.846 (0.712–0.980)	0.001	0.88	87.5	86.7	86.9	87.3	87.1
Post-treatment								
Native thiol	0.583 (0.378–0.789)	0.429	255.6	75.0	73.3	74.0	74.5	74.2
Total thiol	0.588 (0.384–0.791)	0.406	289.5	68.8	73.3	71.0	71.0	71.0
Disulfide	0.512 (0.299–0.726)	0.906	17.0	56.3	60.0	58.0	58.5	58.2
Disulfide/Native thiol	0.675 (0.484–0.866)	0.097	6.96	75.0	66.7	68.5	73.5	70.6
Disulfide/Total thiol	0.475 (0.266–0.684)	0.813	5.38	56.3	60.0	58.0	58.5	58.2
Native/Total thiol	0.525 (0.316–0.734)	0.813	88.56	68.8	60.0	63.2	65.5	64.5
IMA (negated)	0.825 (0.678–0.972)	0.002	−0.83	87.5	80.0	82.0	86.0	83.9
Percentage change (Δ%)								
ΔNative thiol	0.508 (0.300–0.717)	0.937	−7.14	68.8	66.7	67.5	67.8	67.6
ΔTotal thiol	0.413 (0.209–0.616)	0.406	−7.11	62.5	60.0	61.0	61.0	61.0
ΔDisulfide	0.333 (0.140–0.527)	0.114	−21.99	56.3	60.0	58.0	58.5	58.2
ΔDisulfide/Native thiol	0.788 (0.601–0.974)	0.006	−29.40	81.3	73.3	75.8	80.0	78.7
ΔDisulfide/Total thiol	0.333 (0.139–0.528)	0.114	−24.66	62.5	60.0	61.0	61.0	61.0
ΔNative/Total thiol	0.667 (0.471–0.862)	0.114	−4.19	75.0	66.7	68.5	73.5	70.6
ΔIMA (negated)	0.710 (0.516–0.905)	0.046	−2.48	81.3	73.3	75.8	80.0	78.7

Abbreviations: AUC = Area under the curve; CI = Confidence interval; Δ = Percentage change; Disulfide/Native thiol = Ratio of disulfide to native thiol; Disulfide/Total thiol = Ratio of disulfide to total thiol; IMA = Ischemia-modified albumin; Native/Total thiol = Ratio of native thiol to total thiol; NPV = Negative predictive value; PPV = Positive predictive value.

**Table 5:** Univariable and multivariable firth logistic regression analyses for factors associated with pathologic tumor regression.

Variable	Univariable		Multivariable	
	OR (95% CI)	*p*-Value	OR (95% CI)	*p*-Value
Gender	3.54 (1.08–16.03)	0.118	—	
Age	0.68 (0.17–2.80)	0.594	—	
ECOG PS	0.18 (0.02–1.88)	0.122	—	
Diabetes mellitus	1.33 (0.24–7.28)	0.739	—	
Metformin use	4.67 (0.46–47.63)	0.165	—	
Hypertension drug use	1.82 (0.35–9.46)	0.474	—	
Smoking history	1.93 (0.46–8.05)	0.366	—	
BMI	1.09 (0.22–5.45)	0.916	—	
mCCI	0.32 (0.07–1.60)	0.157	—	
Baseline albumin	6.43 (1.03–21.12)	0.025	0.86 (0.08–9.25)	0.165
Clinical T stage	0.03 (0.003–0.32)	0.001	0.70 (0.02–25.4)	0.840
Clinical N stage	2.31 (0.19–28.47)	0.505	—	
EMVI	0.12 (0.06–0.53)	<0.001	0.47 (0.005–43.8)	0.733
CRM	0.16 (0.05–0.39)	<0.001	0.21 (0.02–0.71)	0.003
Baseline CEA	0.88 (0.21–3.59)	0.853	—	
Baseline CA19-9	1.65 (0.36–7.60)	0.519	—	
RT–surgery interval	0.29 (0.05–1.78)	0.166	—	
Pre-treatment native thiol	1.08 (0.18–6.44)	0.930	—	
Pre-treatment total thiol	1.75 (0.25–12.28)	0.570	—	
Pre-treatment disulfide	7.13 (1.36–21.30)	0.010	1.55 (0.38–6.39)	0.540
Pre-treatment disulfide/native thiol	0.47 (0.10–2.12)	0.320	—	
Pre-treatment disulfide/total thiol	2.63 (0.57–12.00)	0.208	—	
Pre-treatment native/total thiol	0.32 (0.07–1.60)	0.157	—	
Pre-treatment IMA	6.00 (1.26–28.55)	0.020	3.63 (1.22–16.20)	0.043
Post-treatment Native thiol	1.09 (0.22–5.45)	0.916	—	
Post-treatment Total thiol	2.17 (0.42–11.30)	0.354	—	
Post-treatment Disulfide	1.47 (0.36–6.05)	0.594	—	
Post-treatment Disulfide/Native thiol	2.57 (0.60–11.06)	0.200	—	
Post-treatment Disulfide/Total thiol	0.67 (0.16–2.77)	0.576	—	
Post-treatment Native/Total thiol	0.86 (0.21–3.58)	0.833	—	
Post-treatment IMA (negated)	8.80 (1.69–45.76)	0.006	—	
ΔNative thiol	1.47 (0.34–6.43)	0.611	—	
ΔTotal thiol	0.61 (0.13–2.79)	0.519	—	
ΔDisulfide	0.42 (0.08–2.11)	0.283	—	
Δ (Disulfide/Native thiol)	1.75 (0.25–12.28)	0.570	—	
Δ (Disulfide/Total thiol)	0.42 (0.08–2.11)	0.283	—	
Δ (Native/Total thiol)	3.79 (1.06–19.05)	0.102	—	
Δ IMA (negated)	6.11 (0.47-38.26)	0.226 *	—	

Abbreviations: AUC = area under the curve; BMI = body mass index; CI = confidence interval; CRM = circumferential resection margin; DCA = decision curve analysis; ECOG PS = Eastern Cooperative Oncology Group performance status; EMVI = extramural venous invasion; IMA = ischemia-modified albumin; mCCI = modified Charlson comorbidity index; OR = odds ratio; RT = radiotherapy; VIF = variance inflation factor. * Fisher’s exact test was applied where applicable. Note: Post-treatment imaging index (post-IMA) was initially considered but demonstrated substantial collinearity with the pre-treatment imaging metric (VIF = 6.8, tolerance = 0.15). Given the small sample size and the penalized likelihood framework of Firth logistic regression, highly correlated predictors were excluded to minimize variance inflation and preserve model parsimony. The final simplified model included variables with acceptable collinearity (VIF < 3) and stable penalized estimates.

## Data Availability

Access to the datasets generated and/or analyzed during the current study is subject to a data-sharing agreement and institutional ethics approval. Data may be obtained from the corresponding author upon reasonable request and completion of the required approval process.
